# Application of a Cloud Video Conference Method for Recruiting Healthy Subjects Into an Early-Phase Clinical Trial During the COVID-19 Pandemic

**DOI:** 10.3389/fpubh.2021.657804

**Published:** 2021-08-19

**Authors:** Zejuan Wang, Aihua Du, Min Li, Siqi Zang, Xiaona Liu, Dan Zhang, Gang Chen, Lina Zhang, Yanan Zhang, Jin Wang

**Affiliations:** Department of Clinical Pharmacology, Aerospace Center Hospital, Beijing, China

**Keywords:** early phase clinical trial, informed consent, video informed process, healthy volunteers, COVID-19 epidemic

## Abstract

**Objective:** Our objective is to explore the effect of applying cloud video conferencing methods to the informed consent process in an early-phase clinical trial during the COVID-19 pandemic.

**Methods:** All participants who intended to participate in the trial were informed via a cloud video conference before signing the informed consent forms (ICF). Then, the attitudes of the participants with the cloud video conference and their understanding of the trial were evaluated using a questionnaire when they visited to sign the ICF onsite.

**Results:** A total of 165 subjects participated in the cloud video conference process, and 142 visited the site to sign and date the ICFs at the center during the appointment time. The survey showed that nearly 100% of the subjects evaluated the video-based informed consent process as very good or good and gave correct answers to questions about the trial. Furthermore, 136 (95.8%) subjects believed that the knowledge about the trial derived via the video-based informed consent process was consistent with the onsite reality, and 139 (97.9%) subjects expressed their willingness to participate in an informed consent procedure undertaken through an online video conference.

**Conclusions:** The video-based informed consent process achieved the same effects as an onsite informed consent process. The former saves time and cost of transportation for the subject and exhibits good public acceptance; especially in light of the COVID-19 pandemic, this process is conducive for reducing the risk of subject infection due to travel and would also help avoid crowding on site.

## Introduction

Informed consent is a fundamental measure to protect the rights and well-being of the subjects in clinical trials. Before starting a trial, researchers must inform potential subjects about the trial, and the potential subjects who agree with the trial procedures are then required to sign an informed consent form (ICF), as per the guidelines of the International Council for Harmonization of Technical Requirements for Pharmaceuticals for Human Use (ICH), 2016. The current widely used approach for obtaining informed consent involves a face-to-face explanation and discussion between potential participants and trained research personnel, usually the doctor. For early-phase clinical trials, the number of healthy participants is usually more than 6 but fewer than 100. However, the investigator needs to screen more volunteers (typically more than 3-fold) to make sure that enough eligible subjects meet the inclusion and exclusion criteria of the trial protocol. Therefore, several potential participants are usually invited to the Phase I clinical trial site on the same day and given detailed explanations for nearly 1 h regarding the informed consent form together as a group of 30 to 50 individuals, followed by a one-on-one discussion with the investigators before signing the ICF. However, gathering the potential participants on-site does not meet the requirements of the COVID-19 pandemic prevention and control. To control and prevent the spread of the virus and ensure smooth conduct of clinical trials and the safety of both health professionals and subjects, a real-time, remote approach telemedicine technology to inform participants should be considered before one-on-one ICF signing is executed. Welch et al. reported in 2016 that the informed consent process embedded into a telemedicine session with remote video conference can eliminate challenges related to the travel and management of personnel at remote sites and improve research recruitment by reducing barriers related to informed consent while preserving human interactions (1). Several studies have examined the use of multimedia interventions to improve potential participants' understanding of a clinical trial (2–6). Showing videos of actual procedures may add to the participants' understanding of what they might be asked to do during a clinical trial (7).

In the era of social media, nearly every adult owns one smartphone and uses the Wechat APP in work and life. Due to the impact of the COVID-19 pandemic on work and life, video conference APP software has risen rapidly in popularity, such as the Tencent Meeting App. This could make the real-time and cloud video conference in the group context feasible, making it possible to avoid gatherings and help control the spread of the virus.

In the traditional informed consent process, the written material provided to potential research participants requires much reading and does not take into account other forms of learning (visual, auditory, or experiential).

The purpose of this study was to examine a new approach for the informed consent process through the use of a real-time cloud video conference technique to test its acceptability to potential healthy subjects, and their comprehension of the real-time cloud video-based informed consent process; the method was applied to an early phase clinical trial during the COVID-19 pandemic, and the overall quality and effect of the video conference-mediated informed consent process was evaluated.

## Methods

### Participants and Methods

This study was conducted in connection with a bioequivalence trial testing oral tablets for Parkinson's disease performed in healthy volunteers in a Chinese Phase I clinical trial center from March 31 to May 12, 2020. It was approved by the ethics committee of Aerospace Center hospital. According to the sample size of the bioequivalence trial, the estimated sample size of this study is about 150, which could meet the requirement of cross-sectional studies.

The third-party company specifically recruited volunteers from the public or their database of history volunteers by posting the recruitment advertisement through Social Media (like Wechat APP) or the internet. Potential participants were invited to join a cloud video meeting hosted by the investigator through the Tencent Meeting App in a group informed consent process when they intended to participate in the bioequivalence trial or just to be of curiosity without any incentives. Applicants that registered and accepted to participate this way subsequently took about 5 min to be trained by one or two recruitment staff on how to use the application software. The research doctor investigator would explain the ICF and relevant information about 1 h into the video meeting for fewer than 60 people due to the capacity of the software requirement. After being given sufficient time to consider the trial, at least overnight, applicants were offered an appointment to visit the study site in person for a face-to-face informed consent process meeting. Participants eligible for the study were asked voluntarily to fill a questionnaire designed to evaluate the effect and quality of the video-based informed consent process.

The study involved the following steps: (a) preparation of the manual of the software application tool (Tencent Meeting App) and training of investigators and registered potential subjects for operating the software; (b) preparation of the newest ICF text and development of the document with pictures and videos to be shown during the meeting; the document covered the trial schedule, related hospital regulations, the seven-step hand-washing method in a graphical form, the protection measures to be taken at home or while traveling in a graphical form, some videos of special procedures and environment introduction, etc.; (c) gathering the potential participants to log in the video conference and testing signal and network quality upon roll call; (d) undertaking of the online informed consent process for about 1 h by one doctor investigator through the cloud video group meeting to explain the above document. As the researcher scrolls through the document and selects text or video, all these materials are updated in real-time on the participant's screen; (e) sends an eICF copy to potential participants and provides an explanation for those with bad bandwidth by phone after the video conference; (f) preparing a self-developed questionnaire by 10 authors regarding the participants' demographic information, evaluation of the effectiveness of the cloud video meeting, and understanding of the ICF of the trial project; (g) the questionnaires were answered by the participants onsite during the appointment time;(h) evaluation of the effect of the remote cloud video meeting through the analysis of the questionnaires when participants visit the study site in person to sign the ICF forms; and (I) holding one-to-one discussion between the investigator and one subject to ensure the effectiveness and quality of the informed consent process. During the one-on-one discussion in person, the investigator asked questions to further assess the effect of the video-based informed consent process and determine if subjects needed to be given further information and explanation about the ICF before the one-to-one signing of the ICF during the consent process in a private room to protect the privacy and confidentiality of their questions.

The questionnaire comprised five sections: (a) an informed consent introduction on the front page (b) sociodemographic characteristics, including age and gender, (c) evaluation of the cloud video meeting effectiveness, including yes or no questions according to the previous experience, perceptional effectiveness, willingness to participate again, satisfaction with the informed explanation of doctors and whether it was easy to understand, multiple-choice or open text questions for the points focused on ICF advantages and drawbacks of video-based informed methods, and one ranked-options question for the overall evaluation of the video-based informed process, and (d) evaluation of the understanding of this ICF by testing the ICF content.

### Data Analysis

Data were entered into an Excel database by one staff member and double-checked by other personnel. Frequency distributions and simple descriptive statistics were used to describe the subjects' general characteristics, to evaluate the video-based informed consent process, and to analyze the participants' understanding of the informed consent form by SPSS (version 16.0). Categorical variables are expressed as numbers and percentiles.

## Results

In this study, 165 subjects participated in the cloud video-based informed consent process together with a medical investigator through the Tencent Meeting App. Subsequently, 142 subjects visited the study site and agreed to complete the questionnaire survey about the cloud video-based informed consent process and sign the written ICF before the trial screening. Participants were 18 to 46 years old with males constituting the majority of the participants at 66.2% (Table 1). Of the 142 subjects, 86 (60.6%) subjects said that they had participated in Phase I clinical trials before; this was the first-time clinical-trial participation for 54 (38.0%) subjects (Table 2).

**Table 1:** General information of the subjects (*n* = 142).

**Variable**	**Category**	**Number**	**Frequency (%)**
Gender	Male	94	66.2
	Female	48	33.8
Age (year)	18–25	26	18.3
	26–30	34	23.9
	31–45	81	57.0
	≥46	1	0.7

**Table 2:** Evaluation of video informed consent process (*n* = 142).

**Item**	**Category**	**Number**	**Frequency (%)**
Have they participated in phase I trials before?	Yes	86	60.6
Is the effectiveness of the video-based informed process consistent with the onsite process?	Yes	136	95.8
Are they willing to participate in the video-based informed again?	Yes	139	97.9
Are they satisfied with the doctors' explanation in this video-informed process?	Yes	142	100
Is the explanation easy to understand?	Yes	142	100
What is your overall evaluation of the video-based informed process?	Very good	126	88.7
	Good	14	9.9
	OK	1	0.7
	Bad	0	0
What points did you focus on the ICF?	Risk	58	40.8
	Drug information	41	28.9
	Blood volume	32	22.5
	Period	40	28.2
	Adverse reaction	74	52.1
	Privacy	46	32.4
	Compensation	37	26.0
What are the advantages of a video-based information method?	Avoid gathering	94	66.2
	Save transportation cost	42	29.6
	Save time	109	76.8
	graphical form	30	21.1
	None	0	0
What are the drawbacks of a video-based information method?	Unstable network	31	21.8
	Noisy environment	13	9.2
	Fast speed	1	0.7
	Network flow limit	10	7.0
	None	58	40.8

### Evaluation of the Video-Based Informed Consent Process

The survey about the effectiveness of the cloud video informed process showed that all 142 (100%) subjects evaluated the effect of the video-based informed consent process to be good or very good without a gender difference in statistics; 136 (95.8%) subjects indicated that the effectiveness of the video-based informed consent process was consistent with that of the onsite informed consent process; 139 (97.9%) subjects expressed the willingness to participate in an online video-based informed consent process again. Among them, 94 (66.2%) subjects believed that the video method could avoid crowding and close contact, 109 (76.8%) agreed that it could save time, 42 (29.6%) subjects thought it could save transportation costs, and 30 (21.1%) subjects indicated that the video-based informed consent process was well-illustrated and easy to be understood. However, 31 (21.8%) subjects indicated that the environment was noisy during the video explanation; only one subject (0.7%) thought that the speed of ICF explanation by the research investigator was fast (Table 2).

### Understanding of ICF

There were 136 (95.8%) subjects who knew the background of the drug being used in this trial, all 142 (100%) subjects knew the schedule and trial period for participating in this clinical trial, and correctly answered the question regarding the admission day date; all subjects appeared to understand the investigation procedure, possible adverse reactions, and related medical measures taken in this study (See Table 3 for details).

**Table 3:** Understanding of informed consent form (*n* = 142).

**Item**	**Number**	**Frequency (%)**
Able to answer the schedule (time and procedure)	142	100
Able to answer the trial duration	142	100
Able to answer the indication of IMP	142	100
Able to answer the background of IMP	136	95.8
Able to answer the adverse reaction	142	100
Able to answer the procedure if symptoms occurred	142	100

## Discussions

The Chinese Good Clinical Practice (GCP) and International Conference on Harmonization of Good Clinical Practice Consolidated Guideline (ICH-GCP) stipulate that the rights, safety, and well-being of trial subjects are the most important considerations and should prevail over the interests of science and society. The ethics committee and informed consent form are the main measures to protect the rights of the subjects (8). At present, the traditional face-to-face informed consent explanation provided on site is still the main mode through which investigators carry out the informed consent process. However, with the increase in the number of clinical trial projects in recent years, the face-to-face informed consent process performed onsite is becoming increasingly challenging due to the high transportation costs for the subjects and the required input of manpower and material resources from research centers. In special circumstances such as the current COVID-19 pandemic, the risk of infection increases due to close contact during travel or the crowding at the site and makes the onsite group informed consent process difficult to be implemented in clinical trial projects.

The “Guidelines for Investigations on the Use of Electronic Informed Consent” issued by the US Food and Drug Administration (FDA) in December 2016 defined electronic informed consent as “electronic informed consent refers to the use of electronic systems and processes that may employ multiple electronic media, including text, graphics, audio, video, podcasts, passive and interactive web sites, biological recognition devices, and card readers, to convey information related to the study and to obtain and document informed consent” (9). Subsequently, Welch et al. (1) proposed the concept of “teleconsent” based on electronic informed consent. By focusing on collecting feedback from original users, they analyzed how to promote the efficiency of remote communications during the electronic informed consent process and provide solutions to the concerns raised by ethics committees regarding the remote process; they also provided important references for clinical trials for the adoption of electronic information methods. Our study showed that most participants (99.3%) who participated in the video-based informed consent process were young subjects whom all expressed positive effectiveness of providing trial-related information via electronic media with very good or good evaluations and only one subject (45 years of age) with an OK evaluation; 139 (97.9%) participants expressed their willingness to participate in an online video-based informed consent process again, with the outcome being consistent with the results of Newlin et al. involving healthy subjects (10). This study was conducted in connection with bioequivalence trial testing. The number and gender ratio would be limited by the criteria of bioequivalence trial and the rate of successful enrollment for volunteers. In terms of results, 94 men and 48 women finally finished the whole process, which met the statistical requirement of this bioequivalence trial in terms of gender distribution. Even the percentage of men (66.2%) is markedly different from the gender distribution in the wider population, there is no statistical difference between gender in the evaluation of overall effectiveness and satisfaction of this new method by the chi-square test. It shows that this video-based informed process could develop or embed in trials irrespective of gender.

During special occasions such as a pandemic, avoiding large gatherings and reducing travel risk are effective measures for preventing the spread of infection. In our survey, 94 (66.2%) participants believed that using the means of a video conference avoided the need for crowding and reduced the risk of virus infection. Therefore, the video-based informed consent process is worth researching and promoting as a supplement or an alternative way to onsite group informed consent meetings. In the traditional informed consent protocol, some subjects may give up visiting the study site because of the long travel distance involved and tight schedule. This study showed that the video informed consent method helped to save time and transportation costs for some participants.

A study by Sonne et al. (7), which also employed a video-assisted process, showed that most participants (78.7%) preferred to obtain information through video-assisted forms compared with paper-based (12.9%) methods, and almost everyone (96.7%) said that the video improved their understanding of the procedures described in the information documents. In this study, the investigators shared the content of the informed consent form on video screens and highlighted the key content of the informed consent form. The screening check-up process, recorded as a small video, was also played to help the subjects intuitively understand the screening process and save time spent on site for the purpose of familiarizing themselves with the surroundings. Measures to prevent and protect subjects from COVID-19 were illustrated in a graphical form. The study showed that the majority of the subjects indicated that the effectiveness of the video-derived information was consistent with that acquired from onsite presentations; some believed that the video was well-illustrated and easy to be understood. In the survey undertaken to evaluate the participants' degree of understanding of the content of the informed consent form, all subjects indicated their satisfaction with the investigators' explanation and thought that it provided a more comprehensive and detailed understanding of the trial process and drug risks. Videos that use pictures and texts tend to catch the participants' attention and improve the subjects' understanding of the content of the informed consent form.

However, the study indicated some drawbacks because video conferencing needs to transmit information through the internet and thus has higher network requirements, which may not be fulfilled in some areas. Some participants (31; 21.8%) indicated that the network was unstable during the video conference. This may be due to weak network signals or the absence of wireless devices in the location where some of the subjects lived. Also, the complete informed consent process took about 1 h, and the subjects were required to stay on the video calls throughout the process; this made it necessary to have a high network flow. According to the questionnaire, some subjects reported that insufficient network flow and the chaotic living environment (of the subject) would have a significant negative, moderating effect on the effectiveness of the video meeting. Therefore, it is recommended to inform subjects in advance regarding the prerequisites for having an interrupted, smooth-running video call, which includes the use of a sufficiently good network or wireless device (which would enable the temporary movement to a place with a better signal).

In the era of social media, nearly every adult owns one smartphone and uses the Wechat APP in work and life. However, video conferences require good network signals and flow, which may be difficult to achieve in remote areas and volunteers with lower income. This study was conducted in a municipal city with good network signals, and the data about drawbacks may therefore be overestimated.

## Conclusions

When the investigator prepared hardware facilities, informed consent, and trial procedures before the video-based informed consent process and the subjects were subsequently trained for using the Tencent Meeting application and participated in the video conference using a good wireless network, the effect of the video-based informed consent process was similar to that of an onsite process. Video and graphic explanations could enhance the subject's understanding of the content of the informed consent and greatly reduce the subject's transportation cost and time, as well as the manpower and material resources of the research center. Under special circumstances such as the COVID-19 pandemic, a video-based approach can reduce the risk of virus transmission compared with the traditional method. Furthermore, the recording of the video conference during the video-based informed consent process can be stored as evidence for inspection later, if necessary. With the continuous development and maturity of electronic technology, video-based informed consent processes could be used alone or as a supplement to the traditional onsite informed consent process.

## Limitations

“Teleconsent” was the term used by the FDA for electronic informed consent. The method reported in this study is different from that of teleconsent since it combines an online (video) and an offline (onsite) process for the acquiring of informed consent. Although it offers many benefits to researchers, some limitations need to be considered before it is utilized for obtaining informed consent during clinical research. First, it requires participants to have access to a computer with a good internet connection. While a large portion of the population will have direct access to broadband at home or the internet through smartphones, not everyone has access to such facilities. Second, the selected remote cloud video meeting should meet the requirement of privacy and confidentiality for participation in the clinical trial, and all the subjects should comply with this requirement so as not to disclose confidential information on trials or subjects.

In addition, this study was conducted in connection with a single trial, and the sample size may thus be limited, and potential participants may have specific characteristics. We also did not investigate the 23 participating individuals who did not visit the study site. There may also be evidence of some educational, financial, or other social sampling biases. As a result, their attitudes about the video-based process in the questionnaire were thus useful, and this could be addressed in a future study.

## Data Availability Statement

The raw data supporting the conclusions of this article will be made available by the authors, without undue reservation.

## Ethics Statement

The studies involving human participants were reviewed and approved by Ethics committee of Aerospace center hospital. The patients/participants provided their written informed consent to participate in this study.

## Author Contributions

ZW, AD, GC, and JW made substantial contributions to the conception and design of this study, to the acquisition of data, and to the analysis and interpretation of the data. ML, SZ, XL, DZ, LZ, and YZ made substantial contributions to the implementation of the study. All authors contributed to the article and approved the submitted version.

## Conflict of Interest

The authors declare that the research was conducted in the absence of any commercial or financial relationships that could be construed as a potential conflict of interest.

## Publisher's Note

All claims expressed in this article are solely those of the authors and do not necessarily represent those of their affiliated organizations, or those of the publisher, the editors and the reviewers. Any product that may be evaluated in this article, or claim that may be made by its manufacturer, is not guaranteed or endorsed by the publisher.
