# Supplementary material for: Application of a Cloud Video Conference Method for Recruiting Healthy Subjects Into an Early-Phase Clinical Trial During the COVID-19 Pandemic
Source: Front Public Health. 2021 Aug 19;9:657804. doi: 10.3389/fpubh.2021.657804 (PMC8416603; doi:10.3389/fpubh.2021.657804)
Supplement: Supplementary file 1 [file Data_Sheet_1.pdf]

On-line Video Informed Consent Research Project Survey

Dear volunteers

Thank you for your time to participate this research project.

This survey is being conducted in support of the on-line video informed consent study. The purpose of this survey is to evaluate the understanding of the whole one-on-one on-line video informed consent procedure. Data collected from this survey will make a better understanding of this project, and provide an evidence of on-line video informed consent in future as reference..

Your participation in this research project is completely voluntary. Your participant privacy and confidentiality will be protected throughout this study. All documentation will be kept strictly confidential. Information from the survey will be coded to preserve participant anonymity and confidentiality. Data collected in support of this research project will be summarized, in anonymous format, in the body of the final report for publication.

Thank you again for your support and cooperation in this survey.

Department of Pharmacology,

Aerospace center hospital

26th, March, 2020

Section 1. General Information

1. Gender male☐ female☐
2. Age 18-25☐ 26-30☐ 31-45☐ ≧46☐

Section 2. Evaluation of video informed consent process

1. Have you participated in any phase I clinical trials before ? Yes ☐ No☐
2. Were you satisfied with doctors' explanation in this video informed process?

   Yes☐ No☐
3. Did you fully understand what the doctor explained?

   Yes, fully☐ Yes☐ Yes, basically☐ No☐
4. Do you think the effectiveness of video-based informed process is consistent with the onsite process?

   Yes☐ Yes, basically☐ No☐
5. Is the doctor's explanation of the informed consent form easy to understand? Yes☐ No☐

6. What points of information did you focus on through this video informed consent process?

   Risk☐ Drug information☐ Blood volume☐ Period☐ Adverse reaction☐

   Privacy☐ Compensation☐

7. Have the doctor fully explained the information what you are concerned? Yes ☐ No☐

8. What is your overall evaluation of the video-based informed process?

   Very good☐ Good☐ OK☐ Bad☐

9. Are you willing to participate the video-based informed process again? Yes ☐ No☐

10. What are the advantages of video-based informed method?

   Save time☐ Save transportation cost☐ Avoid gathering☐ Graphical form☐

   None☐ Others________

11. What are the drawbacks of video-based informed method?

   Unstable network☐ Noisy environment☐ Fast speed☐ Network flow limit☐

   None☐ Others________

Section 3. Trial related information of informed consent form

Please truthfully answer the questions about this trial, if you can't remember them clearly，the doctor would explain again in the face-to-face in person discussion.

1. Are you able to answer the time, schedule and duration of this trial?

   Yes ☐,please go on filling: the admission date______ and the duration days______

   No☐

2. Are you able to answer the indication of IMP?

   Epilepsy☐ Hypotension☐ Parkinson's disease☐ Cerebral infarction☐

3. Are you able to answer the times of oral drug administration during the trial?

   Once☐ Twice ☐ Three times☐ Four times☐

4. Are you able to answer the source and background of IMP? Generic ☐ Original☐

5. Are you able to answer the adverse reaction of the IMP?

   Dizziness☐ Headache☐ Influenza syndrome☐ Discomfort☐ Nausea☐

   Dyspepsia☐ Allergy☐ Angina☐ Arthralgia☐

6. Do you know what to do if you feel uncomfortable?

   Yes☐ No☐

Thank you very much for your filling!
